# Challenging tumor resistance with less toxic, more effective drug combinations: an example from neuroblastoma

**DOI:** 10.1038/s41419-018-0728-1

**Published:** 2018-06-07

**Authors:** Alessandra Gambacurta, Giuseppe Raschellà

**Affiliations:** 1 Department of Experimental Medicine and Surgery, University of Rome “Tor Vergata”, Via Montpellier 1, 00133 Rome, Italy; 2 ENEA Research Center Casaccia, Laboratory of Biosafety and Risk Assessment, Via Anguillarese, 301, 00123 Rome, Italy

Among pediatric tumors, neuroblastoma (NB) accounts for about 5%, but disproportionately causes more than 10% of cancer-related deaths^1^. An international collaborative effort has allowed the subdivision of NB for prognostic and diagnostic purposes into four distinct groups^2^: very low risk, low risk, intermediate risk, and high risk. This division is based on the analysis of various clinical, histological, and biological/genetic parameters including the age at diagnosis, the histological category, the amplification state of MYCN oncogene, and loss or gain of genetic material in specific chromosomes^3^. Although in the past decades, there has been a substantial improvement in the survival of NB patients with very low risk, low risk, and intermediate risk thanks to the application of increasingly accurate multimodal therapies, the treatment of high-risk NB continues to constitute a clinical challenge that urgently needs new therapeutic strategies^3^.

It is worth remembering that among childhood tumors, NB is certainly one of the most characterized in terms of genetic defects. Indeed, the amplification of the MYCN oncogene was among the first genomic alterations that came into use for the definition of a more accurate prognosis and diagnosis. Nonetheless, other factors contribute to the aggressiveness of this disease since a number of high-risk NBs do not show MYCN amplification^1^. More recently, it has been demonstrated by multiple groups independently that the rare familial NB representing about 1–2% of neuroblastomas has a frequent activating mutation of the gene coding for the anaplastic lymphoma kinase (ALK) (reviewed in ref. ^3^). ALK is a tyrosine kinase that had previously been described as an oncogenic driver of other cancers, such as the anaplastic large-cell lymphoma and the non-small-cell lung cancer. Similarly, the ALK mutation plays a major role in the establishment of familial NB^3^. Furthermore, mutations in the ALK gene have also been found in sporadic NBs, widening the possibility of using ALK as a target for therapeutic purposes^3^.

Contrary to the majority of tumors, the p53 tumor suppressor gene occurs in its wild-type form in NB^4^. Potentially, this may constitute an important therapeutic opportunity for the exploitation of p53 proapoptotic activity. Nonetheless, a cytoplasmic sequestration of p53 in non-differentiated NB was highlighted, which constitutes a barrier for its normal role in the nucleus^4^. The transcriptional activity and stability of p53 are inhibited by the MDM2 protein which binds to p53 with high affinity promoting its proteasome-dependent degradation. MDM2 is frequently overexpressed in human malignancies. More than a decade ago, a class of small molecules antagonistic to MDM2 has been characterized^5^. These molecules bind to the p53-binding task of MDM2, thus allowing p53 to play its role in cell cycle arrest, apoptotic induction, and tumor growth inhibition^5^.

A new study by Myazaki et al.^6^ provides evidence that p53 activators can suppress the regrowth of ALK-driven NB, reduce resistance to and enhance efficacy of ALK inhibitors, inducing cell death and preventing tumor relapse more effectively than single-drug treatments.

The greater knowledge of the biological role of ALK has allowed the development of a class of specific inhibitors that have made therapeutic targeting of ALK an achievable goal in the clinic^7^. The use of ALK inhibitors, such as Crizotinib and Alectinib, can delay the progression of ALK-driven cancers, mainly inducing cell cycle arrest, but not cell death and, for this reason, are of limited utility due to the onset of drug resistance, leading to tumor relapse and frequent development of brain metastases^8^. Therefore, novel strategies aimed at preventing resistance are urgently needed. Sequential use of different ALK inhibitors, as well as therapies targeting ALK together with other signaling pathways have been attempted. Indeed, previous studies have shown that ALK inhibition synergizes with chemotherapy or CDK4 inhibition in preclinical models of NB^9^.

The use of ALK inhibitors and chemotherapy seems a more effective clinical strategy than single-drug treatments in NBs with defects in the ALK pathway. In tumors harboring wild-type p53, chemotherapy causes the activation of the p53 pathway which leads to apoptosis, so the activation of this pathway may be an effective way to suppress the growth of ALK-driven NB cells and induce cell death^10^.

Among chemotherapeutic agents, cisplatin, which causes DNA double-strand breaks and activates the p53 pathway, brought about synergistic effects with ALK inhibitors but its genotoxicity can produce risks of adverse reactions and cause chronic diseases in pediatric cancer patients^10^. Nutlin-3a, an inhibitor of the interaction between p53 and MDM2, is a less toxic alternative to cisplatin, although, used alone, it induces cell cycle arrest, but not cell death^11,12^.

**Fig. 1:**
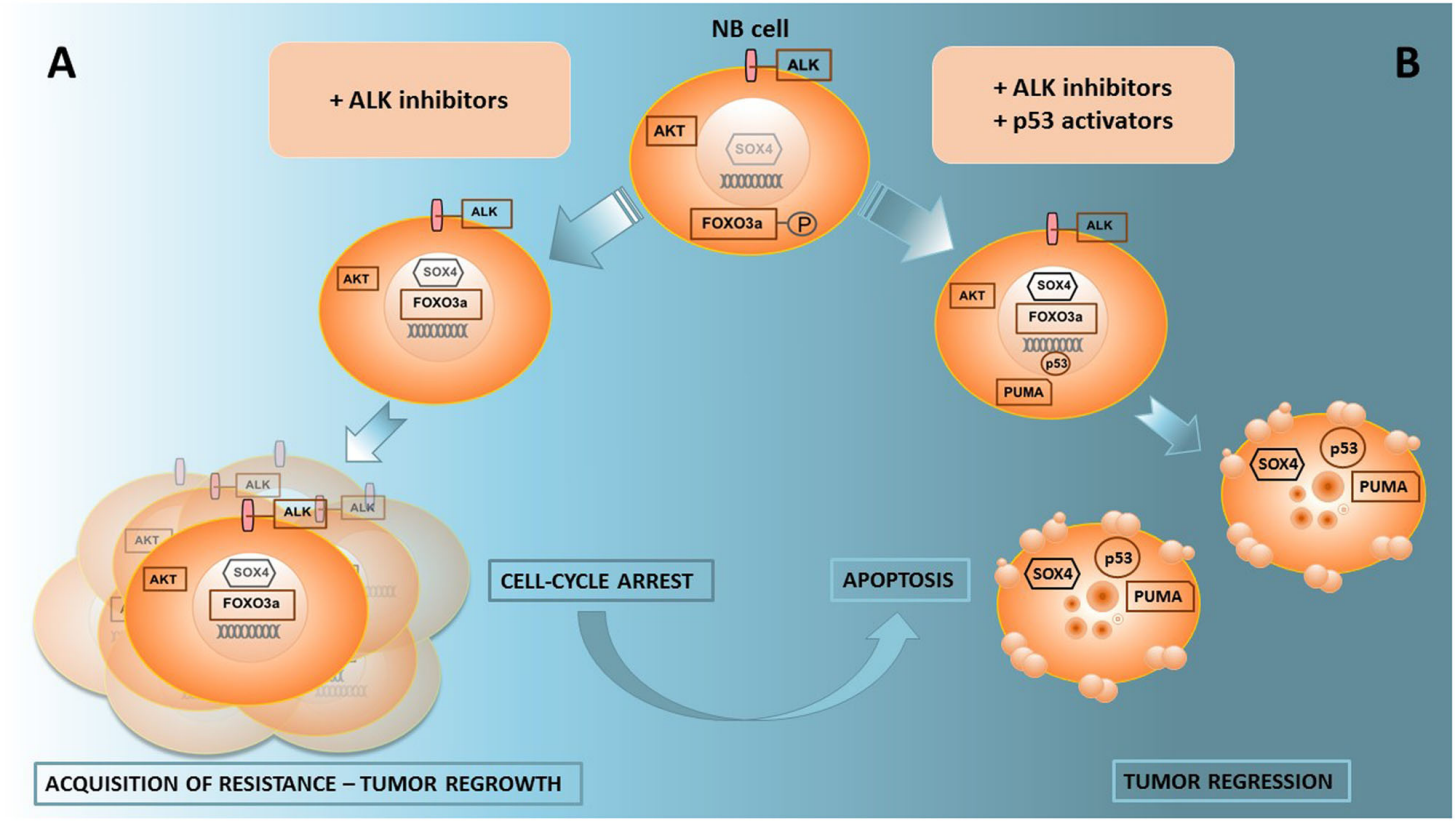
**a** ALK inhibitors induce cell cycle block but not cell death. Tumor cells acquire resistance to ALK inhibitors. **b** Combination of ALK inhibitors and p53 activators, as proposed by Myazaki et al.^6^, causes intrinsic apoptosis through p53-dependent activation of PUMA. SOX4 cooperates with p53 in induction of PUMA. ALK inhibitors mediate the dephosphorylation of transcription factor FOXO3a that migrates to the nucleus to promote SOX4 expression. Combination treatment causes tumor regression

In their paper, Miyazaki and colleagues demonstrate that Nutlin-3a in combination with ALK inhibitors caused cell death in ALK-driven NB cells by triggering intrinsic apoptosis and produced a greater synergistic effect than the combination with cisplatin. ALK inhibitors together with the p53 activator Nutlin-3a are able to induce a switch from cell cycle arrest to apoptosis by inhibition of the ALK–AKT–FOXO3a pathway, leading to an upregulation of the transcription factor SOX4 (Fig. 1). Indeed, ALK inhibitors caused the dephosphorylation of FOXO3a and led to nuclear translocation of the de-phosphorylated FOXO3a in NB cells. Importantly, FOXO3a knockdown partially prevented the SOX4 expression induced by treatment with ALK inhibitors. These data suggest that ALK regulates the expression of SOX4 through the ALK–AKT–FOXO3a regulatory pathway. SOX4 interacts with and stabilizes p53 protein by blocking MDM2-mediated p53 ubiquitination and degradation, promoting the expression of PUMA^13^, the main factor involved in this apoptotic process (Fig. 1).

The work highlights several challenges in the field of new therapeutic approaches, demonstrating that ALK inhibitors, but also other RTK inhibitors, are insufficient to induce cell death. Thus, triggering apoptosis in the tumor cells by using together ALK inhibitors and Nutlin-3a seems a strategy to achieve an effective therapy. If efficacy of inhibitors of other RTKs can be increased by p53 activators, such a treatment could be applicable to other cancers harboring aberrant *RTK* genes. Intriguingly, the authors propose that the combined use of ALKs inhibitors, EGFR inhibitors, and p53 activators can be tested in other malignancies such as lung adenocarcinomas and anaplastic large-cell lymphomas.

Hopefully, the increasing knowledge of the regulators and actuators of the apoptotic switch will likely provide additional therapeutic strategies in ALK-driven cancers with specific reference to targeted drug combinations. The search for the “right” therapeutic agents and the suitable coupling of drugs to promote the apoptotic response in tumors represents a growing research effort aimed at diversifying the therapeutic options and at establishing personalized therapies^14^. Drug repositioning and the thoughtful use of targeted drugs as in the work of Miyazaki and colleagues may constitute a promising strategy to launch more effective therapies for treatment of aggressive tumors such as high-risk NB. In conclusion, the authors not only proposed an effective combination of targeted drugs but also highlight the need to formulate therapeutic regimens that take into account systemic toxicity. This last point is particularly important in NB, where the young age of the patients requires particular attention to the long-term consequences deriving from extremely toxic treatments.
